# Management of cleft lip and palate in adults

**DOI:** 10.4103/0970-0358.57202

**Published:** 2009-10

**Authors:** Jyotsna Murthy

**Affiliations:** Department of Plastic Surgery, Chief Coordinator, Cleft & Craniofacial Centre, Sri Ramachandra University, Chennai, India

**Keywords:** Adult cleft lip, Adult cleft palate, Unoperated adult clefts

## Abstract

**Introduction:**

With advancement of medical services in developed countries and awareness among the patients, it is rare to find an adult with an unoperated cleft lip and palate. However, the scenario is totally different in developing countries. Working as a part of a team in developing country, where co-coordinated team work is primitive, resources to provide treatment are very thin, public awareness of availability of treatment for this anomaly is minimal, the age of patients reaching for primary treatment varies from few days to late forties. Though the aim and aspiration is to provide holistic multidisciplinary care, the priority is getting treatment for all cleft patients. In such situation, the management of cleft lip and palate demands changes of approach, techniques and philosophy.

**Aims and Objectives:**

The deformed anatomy especially the facial bones and dentition is described. Due to well established deformities, the approach for management is individualized. The procedures and modification of procedures has been described.

**Results and Outcome:**

The outcome of the primary repair is adults certainly have less than satisfactory outcome for obvious reasons. The expected outcome and expectation of patients and families following primary surgeries in cleft lip and palate has been discussed. Though all adult patients got some improvement in speech after palate repair, achieving normal speech was difficult. The naso-labial appearance was not perfect, but well accepted by the patients and families. There are many psychosocial problems in these patients, the objective evaluation could not be done due to too many variables. However, primary repair of cleft lip and palate is justified and beneficial for the patients.

## INTRODUCTION

In developed countries, with the advancement of medical services and awareness among patients, it is rare to find adults with un-operated cleft lip and palate. However, the scenario is totally different in developing countries. Working as part of a team in a developing country, where co-coordinated team work is primitive, resources to provide treatment are very thin, public awareness of availability of treatment for this anomaly is minimal and the age of patients reaching for primary treatment varies from few days to late forties. Though the aim and aspiration is to provide holistic, multi-disciplinary care, the priority is getting treatment for all cleft patients. In such situations, the management of cleft lip and palate demands changes the approach, technique and philosophy.

### Facial Bones and Dentition

The growth and deformities of facial bones in a cleft lip and palate patient is uniquely affected by failure of fusion of bones and matrix due to cleft starting from embryonic phase to complete growth. As expected, the facial bones have a normal potential to grow, though mal-positioned in cleft patients. Growth disturbances, especially mid-face retrusion, in cleft lip and palate patients following surgical treatment is a common finding.[1,2] Many details have been written in literature about the growth of facial skeleton in un-operated cleft lip and palate patients. Studies on un-operated adult cleft patients showed that majority of them have normal growth potential without any maxillary retrusion and actual protrusion of maxilla on non-cleft side. The protrusion of maxilla on non-cleft side in unilateral cleft lip and protrusion of pre-maxilla in bilateral cleft lip is mainly because of the absence of normal lip musculature and their forces. In addition, tongue positioning itself into cleft, rotates the alveolus with teeth into more anterior, superior and lateral position. It has showed normal SNA, SNB and ANB angle in un-operated cleft individual as compare to normal control group.[3]

**Figure 1:**
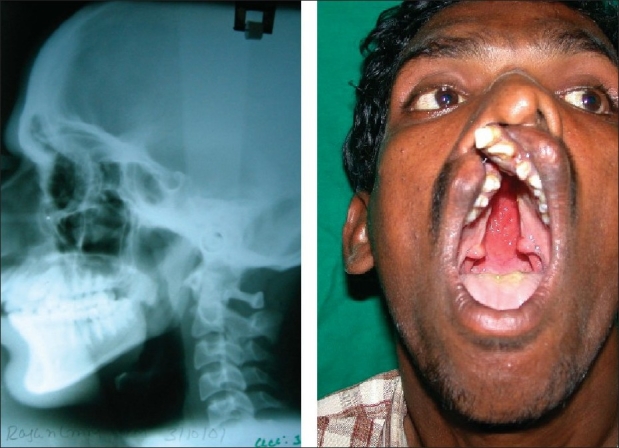
Cephalogram and dental deformities in unoperated cleft patient

Some variations which are secondary to the cleft, though not directly affected by it, have been noticed in mandible. Though mandible has a normal length of ramus and body, the gonial angle is obtuse and mandibular angle to cranial base has increased. These changes put mandible in retruded position with increase in lower facial height. Obviously, due to cleft, there is proclination, rotation and mal-position of anterior maxillary teeth which also alters the mandibular teeth. Often these teeth are permanent and with cleft, compromised bone stock, it is often difficult to realign these teeth orthodontically before surgery [Figure 1]. These skeletal and dental changes demand modification of approach and technique.

### Protocol and plan

Treatment plan is individualized according to age, problem and modified to suite the social condition. Majority of these patients seek quick solutions without frequent visits to the hospital and financial burden of the treatment. In older patients, the surgeon fights with more pronounced soft tissue deformity, wider clefts and unmolding skeletal structure. Functional rehabilitation is the main priority followed by appearance. For all patients older than one year, undergoing primary surgery, the cleft palate is repaired earlier than lip repair. Un-repaired lip forces patients to come back for the surgery and during these few visits, we counsel and treat them for speech and dental deformities. For majority of the patients, the improvement after primary surgery is satisfactory. Primary surgeries provide them enough improvement and majority of the patients do not follow up for secondary correction in spite of counseling and free services which include travel cost. The social circumstance and environmental interaction of these patients is often limited to the family and village.

## SURGICAL PROCEDURES FOR UNILATERAL CLEFT LIP AND PALATE

### Cleft palate repair

The complete cleft of palate was repaired by two flap technique with intravelar veloplasty as described by Sommerlad.[4] In incomplete cleft of secondary palate, Von Langenback technique with intra-velar veloplasty was done. We also preferred alveolar extended palatal flap, which helps to avoid post-alveolar fistula. The specific problems faced in adult cleft palate repair are:

- Very wide cleft
- Adherent muco-periosteal flap due to chronic inflammation following poor hygiene or external agents like smoking or tobacco chewing
- Vertical palate shelves due to constant pressure from tongue

Vertically oriented shelves make paring incision very difficult. We often raise the muco-periosteal flap from the lateral incision and then take paring incision under direct vision from inside out. Adherent periosteum is likely to bleed more giving rise to more possibilities of raising flap in wrong planes. A good nasal layer repair will prevent fistulae like in young children. Cleft palate repair before lip repair allows good exposure to anterior palate region and nasal layer repaired up to the nostril floor.

Post-operatively, all adult patients are started on a semi-solid diet with proper hygiene. Liquid diet like juices and milk shakes are more expensive and unaffordable by many patients. A large quantity of liquid diet is necessary to satiate the hunger in an adult patient. This forced us to change to soft, well-cooked diet immediately post-operatively, from day one, which is much cheaper and easily available.

Aggressive speech therapy is necessary[5,6] but not possible in majority of the patients for logistic reasons which are also the likely reasons for late primary treatment. However, all patients are counseled for home speech therapy. Velo-pharyngeal incompetence correction is done only when patients are motivated and followed up for speech therapy and likely to follow up for speech therapy in future.

### Cleft lip repair

Six months after the palate repair, lip repair is done. Either straight line repair for minor cleft, classical Millard for incomplete, or Mohler's modification of Millard's repair for complete cleft is done. The wide cleft often demands extensive sub-periosteal dissection, up to the zygoma, to mobilize cheek muscles. Primary nose correction is done either as closed nose for minor correction or with the help of marginal incision on the cleft side to position lower lateral cartilage more medially and superiorly with dome to dome suturing to the opposite.

**Figure 2:**
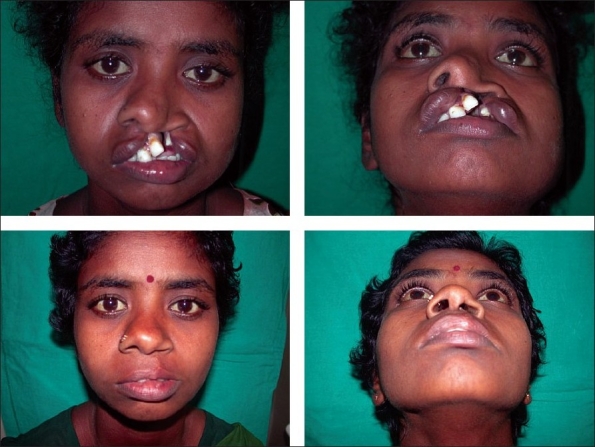
Unilateral cleft lip and palate repair

Too much septal work during primary lip repair is avoided because most of the patients need final rhinoplasty. Mal-positioned and mal-rotated teeth often pose the problems of breaking the mucosal suture line [Figure 2]. However, eventually the mucosa heals. Very rarely, to avoid trauma, we need to put the lining between mucosal suture lines and protruding teeth.[7]

## MANAGEMENT OF UN-OPERATED BILATERAL ADULT CLEFT LIP AND PALATE

In addition to problems faced in unilateral cleft lip repair, bilateral cleft lip has the most noticeable and difficult problem of pre-maxilla. The protruding and twisted pre-maxilla add to the problems of surgical management of complete bilateral cleft lip and palate in older patients. The protruding pre-maxilla, unrestrained by either of the maxillary alveoli, is only attached to nasal septum by a septo-maxillary ligament. In normal children, the cartilaginous septum must slide forward in relation to the pre-maxillary region due to the restraint on pre-maxilla by lip musculature and lateral maxillary segments. In the bilateral cleft, the pre-maxilla is carried forward at the same rate as that of the growing septum to which it is firmly held. The pre-maxilla has only one restraining connection, the vomer. This restrain is realized as a tension between these bones borne by the vomero-premaxillary suture, thus creating the condition for bone formation. Often there is disproportion between the size of the pre-maxilla and the gap where it should lie between the maxillary segments.[8,9]

**Chart 1:**
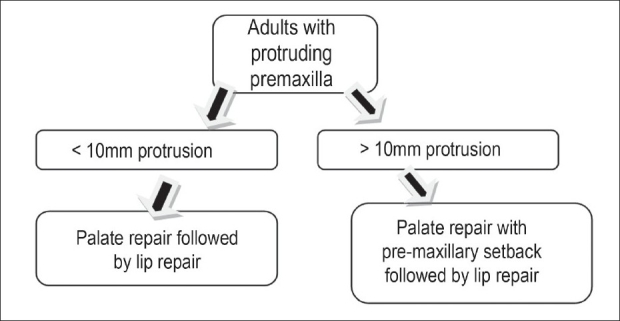
Our plan of managing protruding premaxilla in unoperated adults

Protruding pre-maxilla in older patients coming for the primary treatment is usually protruding and often rotated. This prevents proper bilateral cleft lip repair. Pre-maxilla in adults is unlikely to mould and re-align under the pressure of repaired lips. In adults, when pre-maxilla is protruding more than 8-10mm, compared to the lateral arch and if other condition permits, pre-maxillary set back is planned with cleft palate repair. Our protocol for bilateral adults' cleft lip and palate is as shown in flow Chart 1.

When pre-maxillary is protruding more than 8-10mm, the lip repair becomes very difficult and closure of peri-alveolar oral and nasal layer is compromised. These patients are likely to undergo multiple secondary surgeries which also include repair of oro-nasal fistulae in anterior region of protruding pre-maxilla.[10] To achieve optimal results with fewer surgeries, the present technique of palatal repair and pre-maxillary setback as the primary operation in a single stage was adopted. Padwa *et al*.[11] suggested that a protrusive pre-maxilla could be surgically repositioned after six to eight years without deleterious effects on mid-facial growth. Freihifer *et al*.[12] also noted that the development of maxilla by this age (8 to 13 years) is far advanced and the growth disturbance at this age by pre-maxillary setback has only a relatively restricted negative influence.[13]

### Cleft palate repair

Bilateral cleft palates, like unilateral, are usually very wide with vertically oriented shelves. Vomer is usually unattached to any shelf and hanging in the middle from the cranial base. Often this is complicated by protruding pre-maxilla and very short vomer lying deep away from lateral shelves. The two-flap technique with alveolar extension is routinely done with intra-velar veloplasty. Mucosa on vomer is cut in midline and two flaps were raised. All efforts are made to utilize the vomer flap for nasal lining repair. If pre-maxilla is protruding more than 10mm or severely mal-positioned, not amenable for the orthodontic treatment, set back is done at the same time as palate repair.

### Palate repair with pre-maxillary setback[13]

Mucoperiosteal flaps are raised on both the palatal shelves. The nasal mucosa is separated from the palatal shelves and vomerine flaps raised. Bilateral vomerine flaps are sutured to nasal layer of palatine shelves to repair nasal layer up to the junction of hard and soft palate and intravelar veloplasty carried out.

The required amount of bone is then removed anterior to the vomero-premaxillary suture. The nasal septum is separated from the superior aspect of pre-maxilla and aligned with the lateral maxillary segments. The pre-maxilla is held in a new position and fixation is done with “K” wire. For additional stability we have carried out gingivoperiosteoplasty on the side which is closely aligned and where the alveolar defect was minimal [Figure 3A, 3B]. Simultaneous bone grafting of the cleft alveolus has been avoided in all the patients due to inconsistency of obtaining watertight closure.

**Figure 3A:**
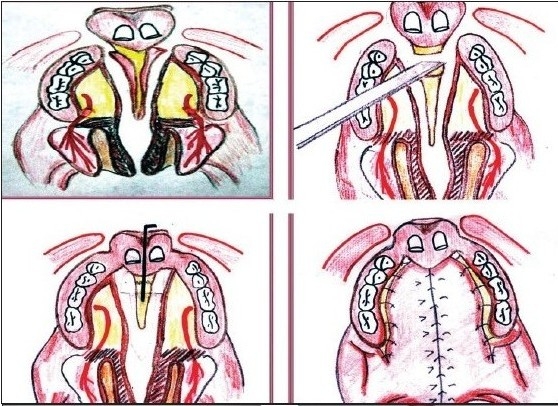
Diagram for pre-maxillary setback with palate repair

**Figure 3B:**
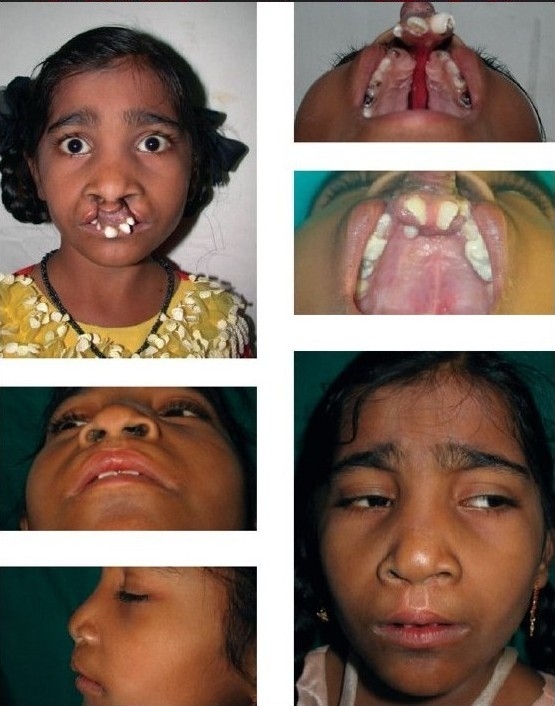
Pre-maxillary setback followed by palate repair

### Bilateral cleft lip repair

Currently, the most commonly used method is Mulliken Modification of Millard's technique. Mulliken emphasizes on narrow philtral column with good muscle repair. His emphasis on “Columella in Nose” is visible in his technique when proper position of cartilages with bilateral marginal incision in nose is done.[15]

**Figure 4:**
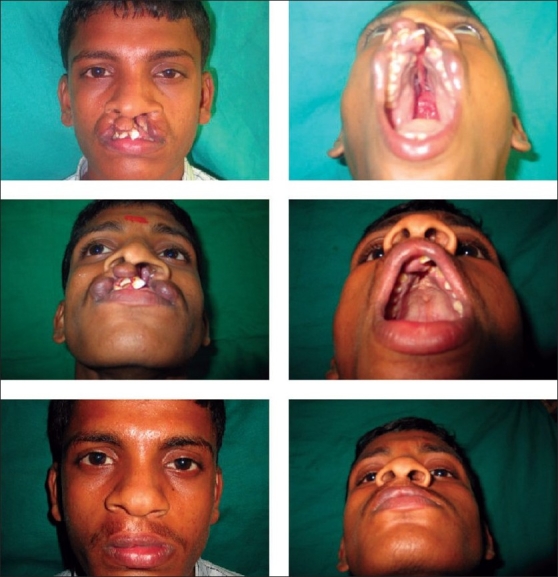
Bilateral cleft lip and palate repair

During bilateral cleft lip repair, philtral column is narrowed to the extent of 8-10 mm in adults depending on the gender of patients. Small strips of one mm each, on either side of prolabium, are de-epithelialised. This helps augment blood supply. Bilateral, wide, sub-periosteal dissection up to zygoma is essential to bring the muscle over the pre-maxilla. Open tip nose correction is often involved dividing the anterior septal artery which is the blood supply for philtral column. And with the above concerned, we do not do open technique for nose correction. Like, in unilateral palate, repair of anterior palate is essential leaving narrow strip of four-five mm on the inferior free border of vomer and pre-maxilla. Once the nasal floor is repaired, alar synching is done to reduce the width of nasal floor and muscle repair is completed. The central tubercle is reconstructed by vermilion with white line from the lateral segment. To avoid whistle deformity of vermilion, separating the muscle from vermilion and suturing muscle is essential. The mucosa of the prolabium attached to pre-maxilla is utilized to deepen the buccogingival sulcus. Noordhoff's[16] stitches are taken for the nose to fix the alar cartilage in new position and obliterate the dead space created by the closed dissection of cartilages [Figure 4].

**Figure 5:**
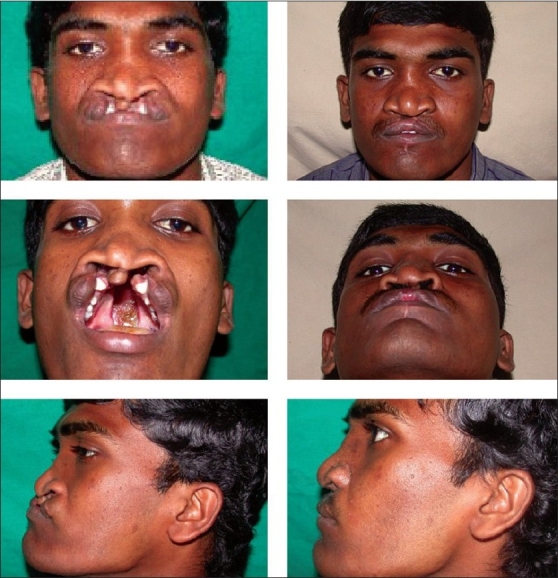
Primary Abbe flap in bilateral cleft lip repair

In a few cases, primary Abbe flap may be necessary due to shortage of tissue in prolabium often found in Median Facial Dysplasia patients [Figure 5].

## FOLLOW-UP MANAGEMENT

Following cleft lip and palate repair, patients are followed up to record the outcome of the primary surgeries. If patients are compliant, the process for VPI correction and orthodontic treatment are started at the same time. Other surgeries like alveolar bone grafting, secondary rhinoplasty are titrated on the demands of patients. In our center, only 25% of them follow up for other surgery, after primary cleft lip and palate repair.

### Hole in one

This terminology is used for the procedure when primary repair of cleft lip and cleft palate has been done simultaneously. It has obvious advantage of less number of surgical procedures, but it has watershed area at the alveolar region. In the Hole-in-one procedure for wide cleft lip and palate, the alveolar region will have only one layer closure which is likely to result in fistula. In very wide cleft, alveolar, an extended periosteal (AEP) flap can be useful. Though we have done it in a few patients, it is not our procedure of choice.

### Primary pharyngeal flap

A large number of patients operated in adulthood have velopharyngeal incompetence (VPI) for many reasons and, logically thinking, primary pharyngeal flap has been suggested and done in a few centres. The speech results following primary pharyngeal flap are not very encouraging. The velopharyngeal port is a complex structure and it is difficult to assess the structural deficiency without palate repair. It is obviously appropriate to do palate repair and give it a chance before re-assessing for VPI correction. This also provides an opportunity to assess if the patient is motivated enough to follow up speech therapy which is essential in most of the adult patients following VPI correction. One series from India when adult un-operated cleft patients were randomized for primary palate repair and primary pharyngeal flap with cleft palate repair showed no difference in speech outcome.[17]

## OUTCOME, CONCERNS AND PROBLEMS OF ADULTS WITH CLEFT AND LATE PRIMARY SURGICAL CORRECTION

Un-operated adult cleft lip and palate is a significant problem in majority of the developing countries. The reasons are obvious: deficient medical services, ignorance and poverty, social and cultural influences and fear of some parents about surgical operations for children! The surgical and rehabilitation care is more complicated with compromised outcome. In addition, majority of them will have psycho-social problems.[18,19]

From our experience, it is interesting to note that patients operated late, were not motivated to follow-up rehabilitation services regularly after primary surgeries. These patients are accepted for their appearance and their defective speech is understood in their immediate environment and the demands from their life are limited.

### Naso-labial appearance

In patients with complete unilateral cleft lip repair with primary nose correction, the outcome of lip repair is acceptable by most of the individuals with cleft. However, the deformities and deviation of nose are severe in complete cleft and we prefer to correct nose at a later date. In our assessment, 80% of patients need formal rhinoplasty in future. Many of them will need extensive dento-alveolar orthodontic treatment to improve dental re-alignment and the facial proportion. Patients who have undergone pre-maxillary setback with palate repair are most likely to have class III occlusion in future. However, this procedure is necessary to avoid anterior fistulae and re-align arches for the alveolar bone graft in future. Class III deformities with good alveolar arches and after alveolar bone graft are more amenable to orthognathic surgery in future.[14]

### Speech outcome

We have analyzed speech outcome of primary palate repair in 131 older patients (over 10 years of age). Pre-operatively, these patients showed mild, moderate and severe articulatory errors of 14%, 48% and 38%, compared to post-operatively - 44%, 48% and eight per cent. Similarly, 64% of patients showed normal or mild resonance post-operatively as opposed to 23% pre-operatively. Nasal emission showed very little improvement, probably due to habituation patterns underlying this problem. The results of the present study showed 55% of the patients within the first three grades of intelligibility post-operatively compared to only 22% pre-operatively. This improvement in intelligibility is attributed to significant improvement in the articulation and resonance postoperatively. All patients showed improvement in all the parameters of speech but very few achieved normal speech.

A study on audiological problem in un-operated cleft palate patients from India showed 76% had mild to moderate conductive deafness.^20^ The established articulation patterns and persistent incompetent velo-pharyngeal port makes speech outcome less than satisfactory.

### Psychosocial problems

Though extensive literature is available for the psycho-social outcome in cleft lip and palate patients, very little is available on the psychological profile of un-operated cleft lip and palate. However, the gravity of these problems is obvious in these patients. Majority of these patients are school drop-outs or have not attended school due to facial deformities and speech problem. They are not acceptable not only by the peers but often by teachers as well! These individuals have greater behavioral problems, more episodes of depression and low self esteem. Teasing has become part of their life and they are unhappy with their facial appearance and capacity to communicate. All these complex psycho-social effects due to un-operated cleft lip and palate make them socially recluse and interact only with the family members.

Speech defects in longstanding untreated clefts are not easily correctable and these have associated life-long impact on the quality of life of patients. Adults with clefts who have speech problems during adolescence and even adulthood generally do not do as well as cleft patients who have not experienced major speech problems. The effects of cultural biases and differences need to be studied in detail for their apparent and considerable influence on patient adjustment, expectations, behavior, and overall ability to accept the condition.

## CONCLUSION

Treating older patients with cleft lip and palate demands a different approach and ingenuity. We need to study carefully the surrounding environment, psychological make-up and motivation of the patient, demands on his/her life and logistical and financial factors to plan their treatment. The treatment may be modified according to the demand of the patients.
